# Human scFvs That Counteract Bioactivities of *Staphylococcus aureus* TSST-1

**DOI:** 10.3390/toxins9020050

**Published:** 2017-02-17

**Authors:** Thunchanok Rukkawattanakul, Nitat Sookrung, Watee Seesuay, Nattawat Onlamoon, Pornphan Diraphat, Wanpen Chaicumpa, Nitaya Indrawattana

**Affiliations:** 1 Graduate Program in Immunology, Department of Immunology, Faculty of Medicine Siriraj Hospital, Mahidol University, Bangkok 10700, Thailand; ruk.thunchanok@gmail.com; 2 Center of Research Excellence on Therapeutic Proteins and Antibody Engineering, Department of Parasitology, Faculty of Medicine Siriraj Hospital, Mahidol University, Bangkok 10700, Thailand; nitat.soo@mahidol.ac.th (N.S.); watee.see@gmail.com (W.S.); wanpen.cha@mahidol.ac.th (W.C.); 3 Department of Research and Development, Faculty of Medicine Siriraj Hospital, Mahidol University, Bangkok 10700, Thailand; nattawat.onl@mahidol.ac.th (N.O.); 4 Department of Microbiology, Faculty of Public Health, Mahidol University, Bangkok 10400, Thailand; pornphan.dir@mahidol.ac.th; 5 Department of Microbiology and Immunology, Faculty of Tropical Medicine, Mahidol University, Bangkok 10400, Thailand

**Keywords:** direct acting anti-TSST-1, human scFv, *Staphylococcus aureus*, superantigen, Toxic shock syndrome (TSS)

## Abstract

Some *Staphylococcus aureus* isolates produced toxic shock syndrome toxin-1 (TSST-1) which is a pyrogenic toxin superantigen (PTSAg). The toxin activates a large fraction of peripheral blood T lymphocytes causing the cells to proliferate and release massive amounts of pro-inflammatory cytokines leading to a life-threatening multisystem disorder: toxic shock syndrome (TSS). PTSAg-mediated-T cell stimulation circumvents the conventional antigenic peptide presentation to T cell receptor (TCR) by the antigen-presenting cell (APC). Instead, intact PTSAg binds directly to MHC-II molecule outside peptide binding cleft and simultaneously cross-links TCR-Vβ region. Currently, there is neither specific TSS treatment nor drug that directly inactivates TSST-1. In this study, human single chain antibodies (HuscFvs) that bound to and neutralized bioactivities of the TSST-1 were generated using phage display technology. Three *E. coli* clones transfected with TSST-1-bound phages fished-out from the human scFv library using recombinant TSST-1 as bait expressed TSST-1-bound-HuscFvs that inhibited the TSST-1-mediated T cell activation and pro-inflammatory cytokine gene expressions and productions.Computerized simulation, verified by mutations of the residues of HuscFv complementarity determining regions (CDRs),predicted to involve in target binding indicated that the HuscFvs formed interface contact with the toxin residues important for immunopathogenesis. The HuscFvs have high potential for future therapeutic application.

## 1. Introduction

Superantigens (SAgs) are proteins produced by some bacterial and viral strains that mediate T cell activation by bypassing the conventional peptide-MHC-II presentation to T cell receptor (TCR) [1]. Instead, intact (unprocessed) SAgs bind directly to MHC-II molecules on the antigen presenting cells (APC)and simultaneously cross-link TCR-Vβ domains shared by about 5%–20% of circulating CD4^+^ and CD8^+^ T lymphocytes [1,2]. T cell stimulation by SAgs is Lck pathway-independent [3], initiated at the Gα11 (a membrane raft-enriched heterotrimeric G-protein) that stimulates PLCβ and PKC to activate mitogen-activated protein kinases (ERKs) causing nuclear translocation of NF-AT and NF-κB and cytokine gene expressions [3]. Massive amounts of cytokines including IL-1β, IL-2, IL-6,TNFα and IFNγ are released from the activated cells into the circulation [1] leading to high fever, rash, skin desquamation (peeling), plasma leakage, obstinate hypotension, and life-threatening multisystem organ failure called toxic shock syndrome (TSS) [2,4]. SAgs potentiate host sensitivity to bacterial endotoxin resulting in TNFα-mediated capillary leakage which is the major contributor of the TSS [5,6]. 

*Staphylococcus aureus* secretes several pyrogenic toxin superantigens (PTSAgs) including toxic shock syndrome toxin-1 (TSST-1) and many enterotoxins [1,7]. TSST-1 is a prototype of group I PTSAgs responsible for most cases of menstrually-related-TSS and a large proportion of non-menstrual cases, i.e., patients with surgical wound and cutaneous infections, osteomyelitis, arthritis, burns, post-partum infection, and barrier contraceptive users [8,9]. Although PTSAgs share nearly identical tertiary structure, their primary sequences are diverse (only 20%–30% identity) and the way they interact with the host receptors (MHC-II and TCR) are different [10,11,12,13,14,15,16]. For examples, *S. aureus* TSST-1 occupies almost one-half of the HLA-DR1 and contact with α-helices of the MHC-II and the bound peptide while *S. aureus* enterotoxin B binds to only one edge of the peptide binding cleft of the DR1 [10]. TSST-1 is encoded by *tst*H gene in the *S. aureus* mobile genetic element [17]. TSST-1 structure and regions that interacted with MHC-II and TCR have been investigated extensively [10,18,19,20]. Mature toxin (194 residues; ~22 kDa) is monomeric in solution and comprises two tightly packed-distinct domains [18,20]. The N-terminal domain (small domain B) acquires α-helix configuration (α1; residues 1–17) that is surrounded by five β-strands (β1-β5; residues 18–89). The C-terminal domain (large domain A) is connected to the N-domain and contains a long α-helix (α2 or the toxin backbone; residues 125-141) packed against fiveβ strands; residues 90–194) that form a β-grasp motif [18,19,20]. N-terminal domain of TSST-1 binds MHC-II, while C-terminal domainis implicated in binding to TCR-Vβ [10,16,18,21].

*Staphylococcus aureus* secretes several pyrogenic toxin superantigens (PTSAgs) including toxic shock syndrome toxin-1 (TSST-1) and many enterotoxins [1,7]. TSST-1 is a prototype of group I PTSAgs responsible for most cases of menstrually-related-TSS and a large proportion of non-menstrual cases, i.e., patients with surgical wound and cutaneous infections, osteomyelitis, arthritis, burns, post-partum infection, and barrier contraceptive users [8,9]. Although PTSAgs share nearly identical tertiary structure, their primary sequences are diverse (only 20%–30% identity) and the way they interact with the host receptors (MHC-II and TCR) are different [10,11,12,13,14,15,16]. For examples, *S. aureus* TSST-1 occupies almost one-half of the HLA-DR1 and contact with α-helices of the MHC-II and the bound peptide while *S. aureus* enterotoxin B binds to only one edge of the peptide binding cleft of the DR1 [10]. TSST-1 is encoded by *tst*H gene in the *S. aureus* mobile genetic element [17]. TSST-1 structure and regions that interacted with MHC-II and TCR have been investigated extensively [10,18,19,20]. Mature toxin (194 residues; ~22 kDa) is monomeric in solution and comprises two tightly packed-distinct domains [18,20]. The N-terminal domain (small domain B) acquires α-helix configuration (α1; residues 1–17) that is surrounded by five β-strands (β1-β5; residues 18–89). The C-terminal domain (large domain A) is connected to the N-domain and contains a long α-helix (α2 or the toxin backbone; residues 125-141) packed against fiveβ strands; residues 90–194) that form a β-grasp motif [18,19,20]. N-terminal domain of TSST-1 binds MHC-II, while C-terminal domainis implicated in binding to TCR-Vβ [10,16,18,21].

TSS management includes supportive and symptomatic treatment. Antimicrobials and surgical debridement to remove the toxin-producing microorganisms are important. Maintaining blood pressure by fluid therapy is necessary [22,23]. Intravenous immunoglobulin (IVIG) confers some benefit to the patients [24].Murine monoclonal antibodies that neutralized endotoxin prevented rabbits from lethal TSS and endotoxin challenge [6]. Symptom severity of TSS was mitigated in a rabbit model after giving a mouse monoclonal antibody that neutralized TSST-1 activities [25]. Rabbit polyclonal antisera against wild type and TSST-1 mutants (G31R and H135A which affected MHC-II and TCR bindings) protected rabbits even when given late in the course of the TSST-1 challenge [26]. In this study, human monoclonal single chain antibodies (HuscFvs) that bound to functionally important residues of TSST-1 were produced by phage display technology. HuscFvs of three phage-transformed *Escherichia coli* clones inhibited TSST-1 mitogenicity (activation of T cell proliferation) and pyrogenicity (induction of pro-inflammatory cytokine gene expressions and the cytokine secretions). The human scFvs have high potential for testing further as a safe, direct acting anti-TSST-1 remedy.

## 2. Results and Discussion

### 2.1. Recombinant TSST-1 and Activities

**Figure 1:**
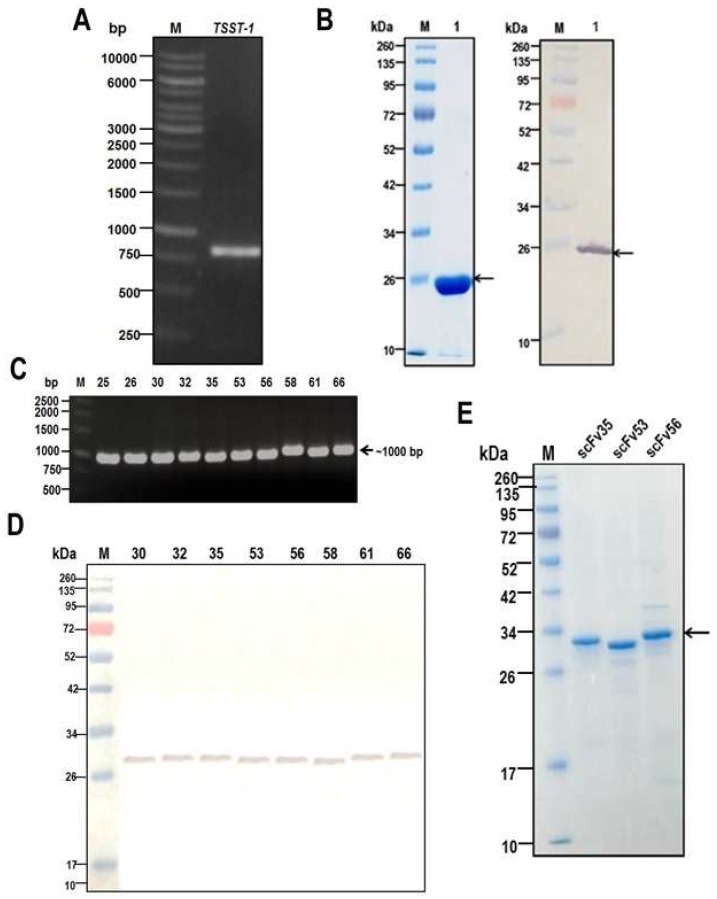
(**A**) Amplicon of TSST-1 gene. (**B**) Stained-SDS-PAGE-separated-rTSST-1 (1 µg per lane) (lane 1 of left panel)and Western blot pattern (lane 1 of right panel). In the Western blotting, mouse monoclonal anti-6× His (AbDSerotec) at 1:3000 was used as the primary antibody; goat-anti-mouse isotype-alkaline phosphatase conjugate (Southern Biotech) at 1:3000 as secondary antibody and BCIP/NBT substrate (KPL). (**C**) Amplicons of *huscfvs* (~1000 bp) from 10 phage-transformed HB2151 *E. coli* clones. (**D**) Binding of HuscFvs in lysates of 8 HB2151 *E. coli* clones to SDS-PAGE-separated-rTSST-1 (1 µg per lane). (**E**) SDS-PAGE-separated- purified and refolded HuscFv35, HuscFv53, and HuscFv56 from transformed NiCo21 (DE3) *E. coli.* M in (**A**,**C**) DNA ladders in base-pairs (bp); M in (**B**,**D**,**E**) protein markers in kDa.

Recombinant pET21a^+^ with TSST-1 gene insert was synthesized (GenScript) and used to transform NiCo21 (DE3) *E. coli*. Amplicon of the gene is shown in Figure 1A. From 1 L culture of the transformed *E. coli* grown under 1 mM isopropyl β-d-1-thiogalactopyranoside (IPTG) induction, 1.46 grams of purified recombinant protein was obtained. The purified preparation revealed only one protein band in SDS-PAGE and protein staining (lane 1, left panel of Figure 1B) and Western blotting (lane 1, right panel of Figure 1B). The LPS content of the purified preparation was 0.15 endotoxin unit (EU)/microgram. One endotoxin unit was approximately 0.1–0.2 ng [27]. Mass spectrometry verified that the recombinant protein was TSST-1 (Table 1). Figure S1A shows codon-optimized nucleotide sequence of the TSST-1 of this study. *BamH*I and *Hind*III restriction sites were placed at the 5′ and 3′ ends of the gene sequence, respectively; a stop codon of the TSST-1 gene was removed from the 3′ end upstream of the *Hind*III site. The deduced amino acid sequence, which has 100% amino acid identity to the TSST-1 of the database (accession J02615), is shown in Figure S1B. 

**Figure 2:**
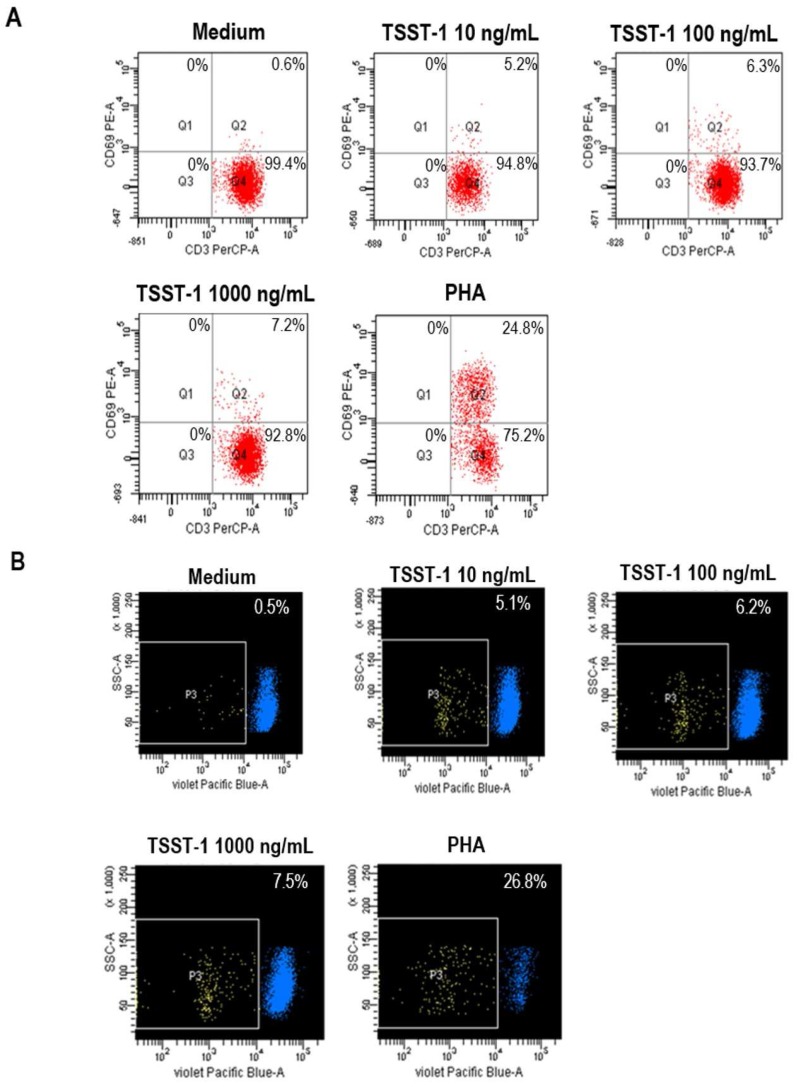
Mitogenicity of the rTSST-1 (ability of the TSST-1 to induce T cell proliferation). (**A**) TSST-1 at 10, 100, and 1000 ng/mL activated T cells to express CD69 (activation marker) by 5.2%, 6.3%, and 7.2%, respectively, compared with the cells in medium alone (0.6%) and PHA (1000 ng/mL)-stimulated cells (24.8%). (**B**) At 72 h after exposure to rTSST-1 (10, 100, and 1000 ng/mL) and PHA (1000 ng/mL), the percentages of proliferated cells were 5.1%, 6.2%, 7.5%, and 26.8%, respectively, compared with 0.5% of the cells in medium alone.

The rTSST-1 at 10, 100, and 1000 ng/mL activated T cells to express CD69 (T cell activation marker) by 5.2%, 6.3%, and 7.2%, respectively, compared with 0.6% of the negative control (cells in medium alone) and 24.8% of the cells stimulated by 1000 ng/mL PHA (pan T cell mitogen) which served as positive control (Figure 2A). At 72 h after exposure to rTSST-1 (10, 100, and 1000 ng/mL) and PHA (1000 ng/mL), the percentages of proliferated cells were 5.1%, 6.2%, 7.5%, and 26.8%, respectively, compared with 0.5% of the cells in medium alone (Figure 2B).

**Figure 3:**
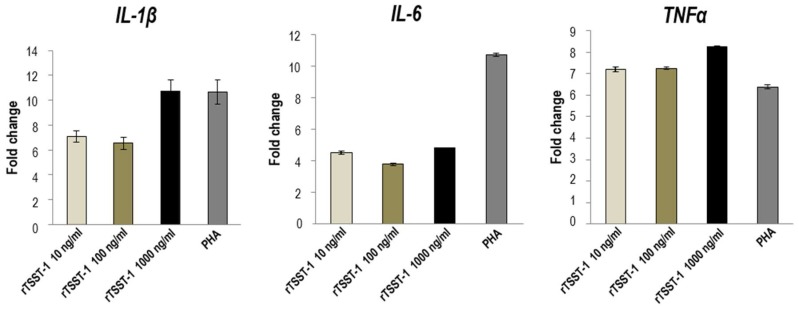
Pyrogenicity of the rTSST-1 (ability to induce stimulated cells to express pro-inflammatory cytokine genes). Fold increase of *IL-1β*, *IL-6*, and *TNFα* expressions in the human PBMCs after stimulation with rTSST-1 at 10, 100, and 1000 ng/mL and 1000 ng/mL PHA (positive control) in relation to non-stimulated cells (negative control).

The rTSST-1 at 10, 100, and 1000 ng/mL were tested for pyrogenicity (ability to induce pro-inflammatory cytokine gene expressions in stimulated cells). Figure 3 shows fold-increase of IL-1β, IL-6, and TNFαgene expressions, respectively, in the human PBMCs after stimulation with rTSST-1 and PHA (positive control) in comparison with non-stimulated cells. Pyrogenicity of the rTSST-1 was not concentration dependent which was conformed to the results reported previously [28].

To exclude the effect of contaminated LPS in the TSST-1 preparation, an experiment in which 1 µg/mL of LPS (Sigma-Aldrich, St. Louis, MO, USA) was used to stimulate the PBMCs was performed. It was found that the LPS induced only 28% of the cell proliferation compared to 100% stimulation by 1000 ng/mL of TSST-1 (Figure S2). Overall results indicated that the bacterially derived-rTSST-1 acquired the inherent activities of the native counterpart. Thus, the active protein was used further.

### 2.2. Production of HuscFvs

Ten colonies of the HB2151 *E. coli* transfected with rTSST-1-bound phages derived from the human scFv phage display library by means of phage bio-panning using the rTSST-1 as bait (see Materials and Methods) revealed amplicons of HuscFv coding genes (*huscfv*; ~1000 bp) (Figure 1C). Only eight clones produced soluble HuscFvs that bound to SDS-PAGE-separated-rTSST-1 (Figure 1D). Even though the HuscFvs bound to the rTSST-1 which was prepared in buffer containing reducing agents (SDS and mercaptoethanol) in the Western blotting, conclusion cannot be made at this stage that the rTSST-1 epitopes are linear sequences [29]. After nucleotide sequencing, three clones (nos. 35, 53, and 56) showed complete HuscFv sequences, i.e., contiguous sequence coding for IgVH, peptide linker (Gly_4_Ser_1_)_3_, and IgVL. Therefore, *huscfvs* of these three clones were subcloned into pLATE52 vector and the recombinant vector was used to transform NiCo21 (DE3) *E. coli* for large scale production of the antibodies. Inclusion bodies were purified from homogenates of the *E. coli* grown under IPTG induction condition and the HuscFvs were refolded. Figure 1E shows SDS-PAGE-separated purified and refolded HuscFvs. The refolded antibodies retained their binding specificity to the TSST-1 coated on the ELISA well surface with and without BSA as determined by indirect ELISA (Figure S3); indicating that the antibodies were refolded properly. The HuscFvs also bound to *S. aureus* enterotoxin A (SEA) (Figure S3).

### 2.3. Presumptive Residues and Regions of TSST-1 Bound by the HuscFvs

TSST-1 3D structure (PDB 2IJO) and modeled HuscFvs (Table S1) were subjected to intermolecular docking. Interactive modes: salt-bridge, hydrogen, hydrophobic, and van der Waals, were selected from molecular dynamic results. The lowest energy scores for TSST-1 complexed with HuscFv35, HuscFv53, and HuscFv56 were −304± 75.4, −243.3± 23.7, and −371.6± 15.3 kcal/mol, respectively. Table 2, Table 3 and Table 4, Figure S4A–C, and Figure S5 show TSST-1 residues and motives that were predicted to form contact interface with HuscFv35, HuscFv53, and HuscFv56. Epitopes of the HuscFv35 and HuscFv56 tend to be conformational, i.e., formed by residues in the separated portions of the TSST-1 molecule that are spatially juxtapose upon the active protein folding, while residues that formed predicted HuscFv53 epitope located mainly between β4 and β5 of the TSST-1 N-terminal domain,suggesting that the epitope might be linear (Figure S5). 

HuscFv35 was predicted to interact with TSST-1 at S1, D4, I6, K7, W12 and S15 of α1-helix, G16 before β1-strand, K67, R68, K71, S72, and Q73 of β4-strand, and β5 Y80 (Table 2). Rabbit immune sera predominantly reacted with residues 1–15 of the TSST-1 N-terminal domain was shown previously to neutralize TSST-1 mitogenicity [30]. Blocking of α1-helix impaired interaction between TSST-1 and MHC-II molecules [30]. S15-G16 peptide bond has been shown to play a role in modulating TSST-1 mitogenicity [31]. A side chain on residue 16 geometrically stabilizes the wild type TSST-1 [32]. G16 (located in the last turn of α1) and S15 were affected when H135 was mutated to alanine and this impaired TSST-1-TCR interaction [30]. Y80W TSST-1 had reduced mitogenicity in a rabbit model [33]. TSST-1 with R68A and S72A mutations failed to activate T cells carrying Vβ2 TCR [34]. Functions of K67, K71, and Q73 are elusive. Results of computerized simulation suggested that HuscFv35 should be able to reduce TSST-1 mitogenicity by interfering with TSST-1 binding to TCR-vβ and cause reduction of TSST-1-mediated cellular cytokine release by interfering with several important residues of the toxin.

By the *in silico* docking, HuscFv53 contacted with D18 (before β1-strand), D39 (before β3-strand), and R68; K71 and H74 (β4-strand); S76 and E77 (before β5-strand); and Y80 (β5-strand) of the TSST-1 N-terminal domain (Table 3, Figures S4B and S5). R68A rendered TSST-1 inability to bind to the HLA-DR2 [34]. Y80W TSST-1 had reduced mitogenicity in a rabbit model [35]. Functions of other residues predicted to form contact interface with the HuscFv53 are unknown. Based on the computerized results, the HuscFv53 should not be as effective as the HuscFv35 in mitigation/amelioration of the TSST-1 bioactivities.

HuscFv56 was predicted to interact with TSST-1 K7, L10, D11 and S15 of α1-helix; S17 and D18 before β1-strand; T19 and F20 of β1-strand; D39 before β3-strand; N65, R68, K71, S72 and H74 of β4-strand; Y80 of β5-strand; K114, Y115, and P117 before β8-strand; K118 and F119 of β8-strand; E132, H135, T138, Q139 and I140 of α2-helix; and R145 before β9-strand (Table 4, Figures S4C and S5). Importance of TSST-1 R68 and S72 on the TCR-Vβ binding and N-terminal residues 1–15 (which include K7, D11, and S15) and Y80 on T cell mitogenicity has been mentioned above. H135 and Q139 on the α2-helix have been shown to be important for the TSST-1 superantigenicity [18,32,35,36,37]. Previous data indicated that H135A TSST-1 mutant possessed only 5%–10% mitogenicity of the wild type [35]. H135 and Q139 are individually critical for functional activity and direct interaction of TSST-1 with TCR-vβ [37]. TSST-1 with H135 mutated to alanine had abolished capacity to induce TNF-α and IL-6 mRNA expressions and protein production [35]. Y115 is a pronounced inducer of IL-6 and TNFα as well as IL-8 [36]. Y115A mutant of TSST-1 had much reduced mitogenicity on T cells and did not express significant toxicity in the rabbit model of TSS [35]. Because the HuscFv56 formed interface contact with several important TSST-1 residues, this antibody should be able to neutralize the TSST-1 activities.

### 2.4. Inhibition of TSST-1 Activities by HuscFvs

**Figure 4:**
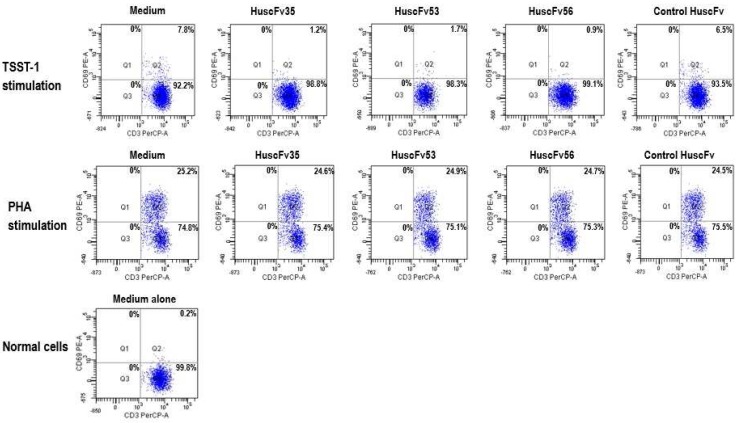
Percent activated CD3^+^CD69^+^ among the CD3^+^ cells exposed to TSST-1 (1000 ng/mL) after treatment with individual HuscFvs (4 µg), control HuscFv (4 µg), and medium for 24 h. The percent TSST-1-activated T cells (7.8%) was markedly reduced after exposure to HuscFv35 (1.2%), HuscFv53 (1.7%), and HuscFv56 (0.9%). The control HuscFv had some inhibitory activity (placebo effect) on the TSST-1-activated cells (6.5%). The TSST-1-specific-HuscFvs did not affect the PHA-stimulated cells indicating their target specificity.

Experiments were performed to verify the computerized intermolecular docking results. TSST-1-mediated 7.8% T cell activation (Figure 4). After treatment with HuscFv35, HuscFv53, and HuscFv56, percent activated T cells were reduced to 1.2%, 1.7%, and 0.9%, respectively (Figure 4). The inhibitory activities of the HuscFv35 and HuscFv56 were higher than the HuscFv53 which conformed to the computerized prediction that the former interacted with several TSST-1 residues important for superantigenicity. The HuscFvs did not cause percent CD69^+^ cell reduction among the PHA-exposed-CD3^+^ cells, indicating their target specificity. Control HuscFv showed modest inhibitory (placebo) effect on the percent CD69^+^ cells (6.5%). Normal PBMCs contained 0.2% CD3^+^CD69^+^ cells.

**Figure 5:**
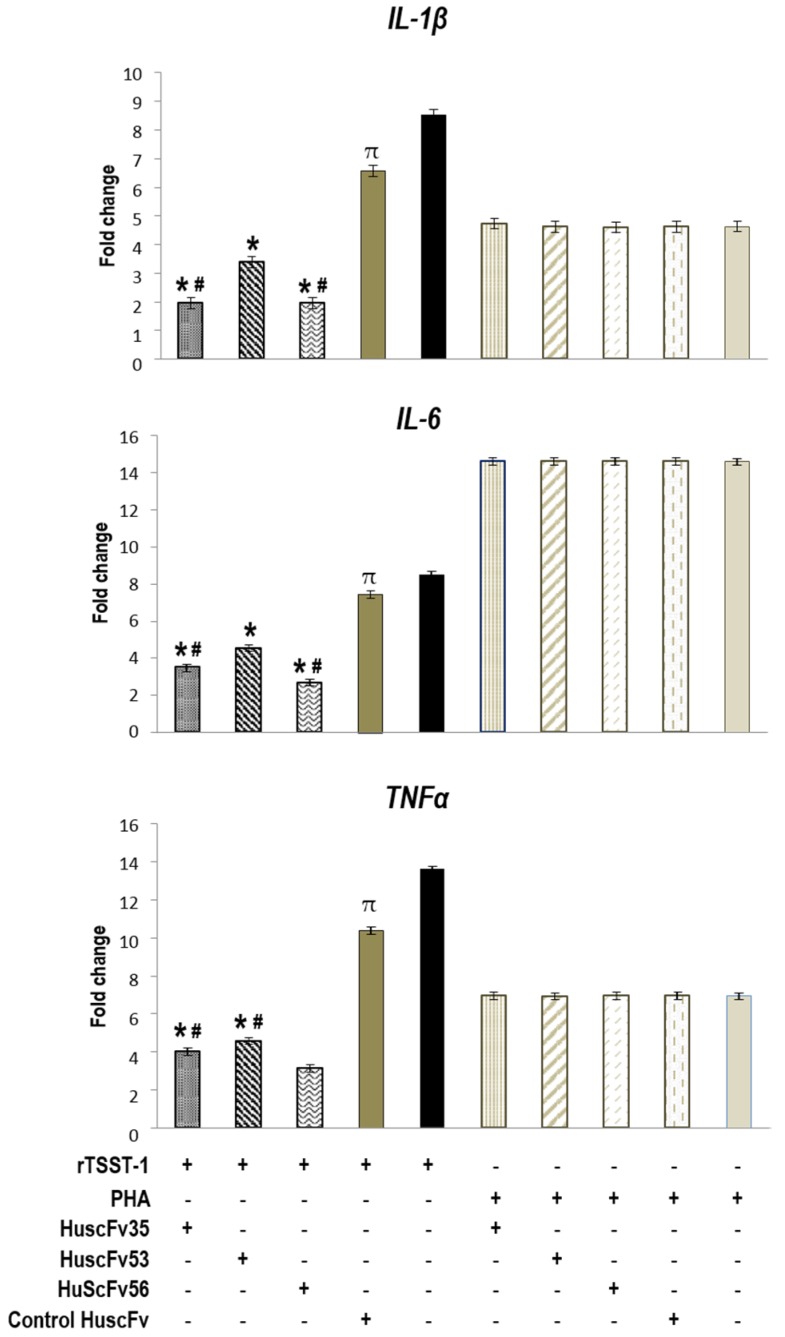
Fold change of IL-1β, IL-6, and TNFα gene expressions in human PBMCs that had been exposed to 1000 ng/mLof TSST-1 and treated with 4 µgof HuscFv35, HuscFv53, and HuscFv56 and controls for 24 h. *, significantly different from (lower than) both controls (*p* < 0.05); #, significantly different from HuscFv53-treated, TSST-1-exposed cells (*p* < 0.05);π, significantly different from TSST-1-exposed cells in medium alone, indicating a placebo effect of the control HuscFv.

**Figure 6:**
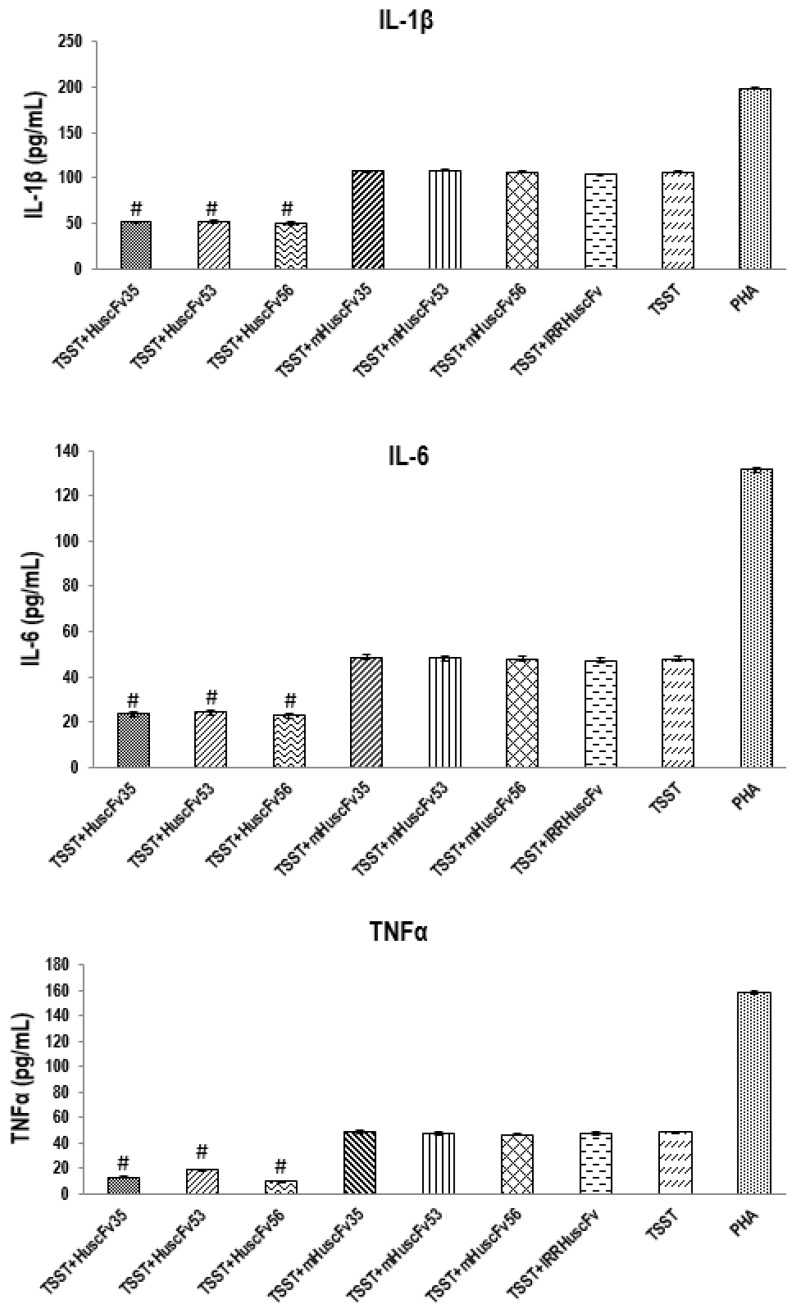
Levels of IL-1β, IL-6, and TNFα cytokines in culture supernatants of PBMCs exposed to 1000 ng/mL of rTSST-1after treatment with 4 µg of TSST-1-bound-HuscFvs, 4 µg CDR mutated-HuscFvs, negative control (TSST-1-exposed cells in medium alone), and positive control (cells stimulated with 1000 ng/mL PHA). #, significantly lower than the groups treated with mHuscFvs and TSST-1- and PHA-stimulated cells.

TSST-1-exposed-human PBMCs added with the HuscFvs had markedly reduced cytokine gene expressions (Figure 5) and the respective cytokine levels (Figure 6), compared to the control HuscFv-treated and non-treated cells (*p* < 0.05). Both HuscFv35 and HuscFv56 performed better than the HuscFv53. Noantibodies had an effect on the PHA-exposed cells, indicating that the HuscFv inhibitory effect on the TSST-1 pyrogenicity was target specific. The data obtained from PBMCs exposed to rTSST-1 and PHA of Figure 5 do not fit with the data of Figure 3. The reason should be that the experiments were performed on blood samples taken a few months apart, although from the same blood donor. However data of duplicate experiments performed on blood samples taken from the blood donor one or two days apart were not statistically different, as shown by the small error bars of both Figures.

**Figure 7:**
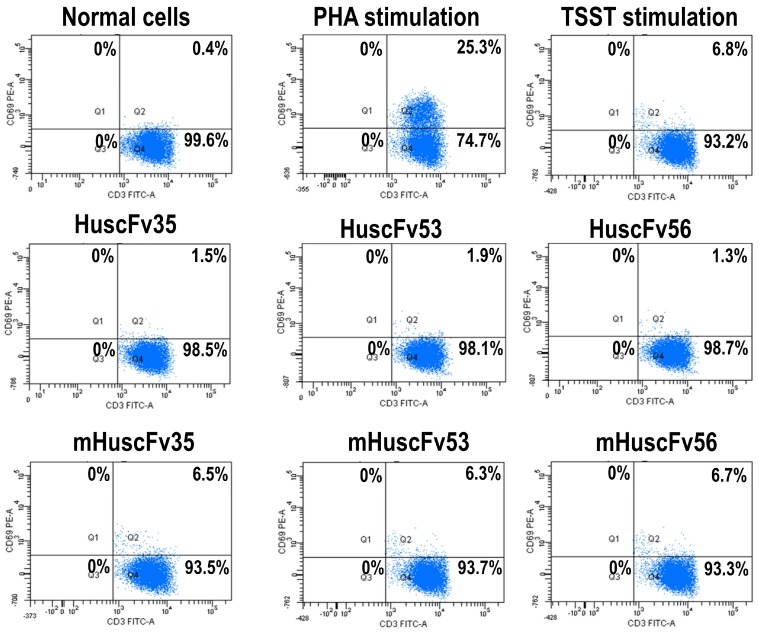
Percent activated T cells (CD3^+^CD69^+^) among the TSST-1-exposed PBMCs after treatment with HuscFv35, HuscFv53, and HuscFv56, mutated-HuscFvs (mHuscFv35, mHuscFv53, and mHuscFv56), control HuscFv, and medium. The mutated-HuscFvs could not reduce TSST-1 mitogenicity on the human PBMCs (percent activated T cells were not different from the TSST-1-exposed PBMCs cultured in the medium alone).

Table 2, Table 3 and Table 4 provide information on the amino acids, their positions, CDRs, and domains of the scFvs that have been predicted to involve in target binding. In order to demonstrate the relevance of the predicted HuscFv residues that formed interface contact with the TSST-1, many of the HuscFv residues which their side chains interacted with TSST-1 were substituted by alanines (marked red in Table 2, Table 3 and Table 4). The mutated residues for HuscFv35 were Y27A of VH-FR1; D31A of VH-CDR1; H100A, Q101A, and D108A of VH-CDR3; T165, N166A and Y168A of VL-CDR1; Y185A of VL-FR2; and T192A of VL-FR3; for HuscFv53 were R31A of VH-CDR1; T52A and D57A of VH-CDR2; Y168A of VL-CDR1; and K186 of VL-CDR2; and for HuscFv56 were S31A of VH-CDR1; E55A and E59A of VH-CDR2; Y101A, Y102A, and R104 of VH-CDR3; Y165A of VL-CDR1; Y182 of VL-FR2; N186 of VL-CDR2; and S225 and Y229 of VL-CDR3. The inhibitory activities of the wild type HuscFvs on the TSST-1-mediated cell proliferation and pro-inflammatory cytokine production were abrogated after the residue mutations, i.e., the mHuscFvs could not reduce mitogenicity and pyrogenicity of the TSST-1, as shown in Figure 7 and Figure 6, respectively.

Antibodies of heterologous source have been shown to mitigate symptom severity and rescued animals from the TSS-mediated lethality [30,31]. Treatment of human TSS cases is usually performed in the intensive care unit and includes supportive and symptomatic measures, removal of bacteria producing the causative toxin as well as infusion of IVIG thought to contain antibodies to bacterial endotoxin. However, passive immunization and immunotherapy by using homologous (human) antibodies directed to the TSST-1 functionally critical residues has never been performed. Human scFvshave potential applications for immunotherapy of diseases [38,39,40]. Thus, the fully human scFvs produced in this study, especially the HuscFv35 and HuscFv56 have high potential for testing further as a safe, direct acting anti-TSST-1 remedy.

## 3. Materials and Methods

### 3.1. Recombinant TSST-1 (rTSST-1) Production

TSST-1 gene was retrieved from GenBank no. J02615. Synthetic TSST-1 gene sequence with stop codondeletion and *BamH*1 and *Hind*III restriction sites incorporation at the 5′ and 3′ ends, respectively, was inserted into pET21a^+^ DNA (GenScript). The recombinant plasmid was used to transform NiCo21 (DE3) *E. coli* by means of a highly efficient transformation protocol (New England Biolabs, UK). Appropriately transformed *E. coli* colony was grown in LB-A broth containing 1 mM IPTG and the 6× His-tagged-rTSST-1 was purified from the bacterial lysate by using Ni-NTA resin (Invitrogen, Waltham, MA, USA).

### 3.2. Mitogenic and Pyrogenic Activities of rTSST-1

Because activated T cells expressed surface CD69 molecules [41]; thus, mitogenicity testing of the rTSST-1 was performed by detecting percentages of CD3^+^CD69^+^ in human PBMCs after exposure to the toxin and controls. Human PBMCs (3× 10^5^ cells/well) were cultured in 48-well round-bottom tissue culture plate (Corning) in RPMI-1640 medium (Gibco^TM^) supplemented with 10% fetal bovine serum, 2 mM l-glutamine, 100 units/mL penicillin, and 100 µg/mL streptomycin (complete medium) at 37 °C in 5% CO_2_ atmosphere. Various concentrations of rTSST-1 (10–1000 ng/mL) were added appropriately to the cells. Positive control was cells stimulated with 1000 ng/mL phytohemagglutinin (PHA) (Sigma) which is a pan T cell mitogen and negative control was cells in culture medium alone. After 24 h, cells were washed twice with cell washing/blocking reagent (1% heat-inactivated normal serum in PBS), re-suspended in fresh culture medium, added with anti-CD3-PerCP and anti-CD69-PE, and subjected to FACScan flow cytometry with BD Diva software for data acquisition and analysis. Lymphocyte population was identified using forward and side scattered property. T cell population was identified by cells that were CD3^+^. The percentage of activated T cells (CD3^+^CD69^+^) was determined from an upper-right quadrant (Q2) where as Q4 was CD3^+^ cells. The Q1 and Q3 were non-activated cell population (CD3^-^CD69^-^). Results are expressed as percentages of CD3^+^CD69^+^ cells.

For testing rTSST-1-mediated cell proliferation, human PBMCs were incubated with violet CellTrace^TM^ for 20 min in complete medium before stimulating with rTSST-1 as above. Similar controls were included. After 72 h, cells were washed, stained with anti-CD3-PerCP, and subjecting to FACScan flow cytometry. Results were expressed as percentages of violet/pacific blue stained-CD3^+^ cells.

Pyrogenicity of rTSST-1 was tested. Human PBMCs (1× 10^5^ cells/well) were cultured and stimulated with various concentrations of rTSST-1 as above. Similar controls were included. After 24 h, total RNAs were extracted from cells in individual wells. Expressions of pro-inflammatory cytokine genes including *IL-1β*, *IL-6*, and *TNFα* were determined by quantitative real-time RT-PCR (qRT-PCR) using primers listed in Table S2. One microliter cDNA(50 ng) and 100 nM each PCR primer was put in SYBR Green Master Mix (Applied Biosystems) and subjected to PCR reaction: 95 °C, 10 min then 36 cycles of denaturation at 95 °C for 30 s, annealing at 60 °C for 1 min, extension at 72 °C for 30 s, and hold at 72 °C for 5 min in Strategene Mx3005P QPCR System (Agilent Technologies). Data were analyzed using MxPro QPCR software. β-actin gene was used for normalization. Levels of the pro-inflammatory cytokines in cell culture supernatants were determined by using ELISA kit (Thermo Fisher Scientific, Waltham, MA, USA).

### 3.3. Production of TSST-1-bound HuscFvs

Human scFv phage display library used in this study was constructed previously [42]. Briefly, cDNAs were prepared from mRNAs of peripheral blood lymphocytes of multiple human blood donors and used as templates for amplification of immunoglobulin VH and Vκ coding sequences by PCR. The oligonucleotide primers used for the PCR were human degenerate primers designed from all families of human immunoglobulin variable sequences [42]. The PCR amplified *vh* and *vl* sequences were ligated randomly via a polynucleotide linker (coding for (Gly_4_Ser)_3_) to generate a repertoire of *vh*-linker-*vl* sequences or *scfv* sequences. The *scfvs* were ligated with pCANTAB5E phagemid DNAs and the recombinant phagemids were used to transfect TG1 *E. coli*. After growing the recombinant phagemid-transformed *E. coli* in the presence of helper phage (M13KO7), complete phage particles which displayed human scFvs as fusion proteins with the phage coat protein (p3) and also carried the respective *scfvs* in the phage genomes could be obtained from the *E. coli* culture supernatant.

HuscFv-displayed phage clones that bound to the rTSST-1 were fished-out from the library using the recombinant protein as bait in the biopanning process [42]. The phage library was added to an ELISA well pre-coated with 1 µg of purified rTSST-1 and the plate was incubated at 37 °C for 1 h. Unbound phages were removed by washing with buffer and a log phase-grown HB2151 *E. coli* culture was added to the well containing the antigen-bound phages.The phage transformed bacterial coloniesthat grew on selective agar plates after overnight incubation were screened for the HuscFv genes (*huscfvs*) by PCR [42]. The *huscfv*-positive clones were grown in 0.2 mMIPTG-conditioned broth to induce HuscFv expressions. Binding of soluble HuscFvs in the bacterial lysates to the SDS-PAGE-separated-rTSST-1 were tested by Western blotting. Nucleotides of the *huscfvs* coding for rTSST-1-bound-HuscFvs were sequenced, deduced, and canonical complementarity determining regions (CDRs) and immunoglobulin framework regions (FRs) were determined using the IMGT^®^ Information System [43].

For large scale production of HuscFvs, *huscfvs* of HB2151 *E. coli* clones of interest were subcloned from the phagemids to pLATE52^TM^ expression vector by using ligation independent cloning (LIC) system (Thermo Fisher Scientific). The pLATE52-*huscfv* plasmids were used to transfect NiCo21 (DE3) *E. coli.* Selected transformed bacterial colonies were grown under IPTG induction; the bacterial pellet was suspended in BugBuster^®^Protein Extraction buffer (5 mL/g bacterial wet weight) and kept at 25 °C with agitation. The preparation was added with Lysonase^TM^ Bioprocessing reagent (10 μL/g of bacteria) and agitated further for 20 min. *E. coli* inclusion body was harvested by centrifugation, washed twice with Wash-100 Solution (50 mM sodium phosphate buffer, pH 8.0; 500 mM NaCl; 5 mM EDTA; 8% (*w*/*v*) glycerol; 1% (*v*/*v*) TritonX-100); twice with Wash-114 Reagent (50 mM Tris buffer, pH 8.0; 300 mM NaCl; 1% (*v*/*v*) Triton X-114), and once with Wash-Solvent (50 mM Tris buffer, pH 8.0; 60% (*v*/*v*) isopropanol) by shaking the preparation vigorously followed by centrifugation. For HuscFv refolding, the inclusion body was solubilized (*w*/*v*) in buffer (50 mM CAPS, pH 11.0; 0.3% (*w*/*v*) N-lauryl sarcosine; 1 mM DTT) and kept at 4 °C for 16 h. The preparation was loaded into the Slide-A-Lyzer^®^ 2K Dialysis Cassettes G2 (Thermo Fisher Scientific), dialyzed at 4 °C with slow stirring against refolding buffer (20 mM imidazole, pH 8.5 supplemented with 0.1 mM DTT), filtered through 0.02 µm low protein binding Acrodisc^®^ syringe filter (Pall, Port Washington, NY, USA), and kept in water-bath at 30 °C for 2 h before adding with 60 mM trehalose. Protein content was determined. The refolded-HuscFvs were retested for binding to rTSST-1 by indirect ELISA.

### 3.4. Computerized Simulation for Determining Interactive Residues between TSST-1 and HuscFvs

TSST-1 3D structure was retrieved from RCSB PDB 2IJO. The *huscfv* 3D structures were modeled by the I-TASSER server [44,45]. The I-TASSER-predicted structures were further refined [46,47] and improved to near native states on the automated ClusPro 2.0 antibody-protein docking server. The models from the docking were simulated with NAMD Molecular Dynamics [48]. The TSST-1-HuscFv complexes were built and visualized by using PyMol software (PyMol Molecular Graphics System, Version 2 edu, Schrodinger, LLC).

### 3.5. Preparation of Mutated-HuscFvs (mHuscFvs)

Gene sequences coding for HuscFvs which side chains of their residues interacted with TSST-1 (data from molecular dynamics) were substituted by alanines and synthesized (Integrated DNA Technologies, Coralville, IA, USA). The DNA fragments were cloned into pLATE52 and the recombinant vector was used to transform NiCo21 (DE3) *E. coli*. The HuscFvs were prepared from appropriately transformed *E. coli* as for the wild type HuscFvs. The mutated HuscFvs (mHuscFvs) were tested for their ability to inhibit TSST-1 activities (mitogenicity and pyrogenicity).

### 3.6. HuscFvs-mediated Inhibition of TSST-1 Activities

For inhibition of TSST-1-mediated T cell activation by the HuscFvs, human PBMCs were added with mixture of rTSST-1 and HuscFvs/mHuscFvs or control HuscFv and kept for 24 h. TSST-1-stimulated cells without antibody treatment, PHA-stimulated cells treated similarly with TSSTS-1-bound-HuscFvs, and normal cells in medium were included in the experiment. After washing, cells were labeled with CD3-PerCP and CD69-PE and analyzed by FACScan Flow cytometry. Total viable lymphocytes were gated by SSC and FSC and CD3^+^ cells were gated for CD69^+^ cells. Results were expressed as percentages of CD3^+^CD69^+^ cells. 

For inhibiting rTSST-1 pyrogenicity by the HuscFvs/mHuscFv, human PBMCs (5× 10^4^ cells/well) in complete medium were added with 1000 ng/mL rTSST-1. The TSST-1-stimulated cells were treated either with TSST-1-bound-HuscFvs/mHuscFv, control/irrelevant HuscFv (did not bind to TSST-1), or medium alone. The antibody:TSST-1 molar ratios were 4:1 (optimal from titration). Triplicate wells were set for each treatment. Cells added with 1000 ng/mL PHA with and without HuscFv-treatments, and cells in medium alone (normal cells) were included. After 24 h, total RNAs were extracted from cells in individual wells and quantified by using NanoDrop instrument. Complementary DNAs were synthesized (RevertAid First Strand cDNA Synthesis kit) and used as templates for quantification of mRNAs of pro-inflammatory cytokines including TNF-α, IL-1B, and IL-6. The quantitative real-time PCR primers for the mRNA quantification are listed in Table S2. β-actin gene was used for normalization. Moreover, cell culture supernatants in all wells were collected and the levels of the pro-inflammatory cytokines were measured using commercial ELISA kits (Thermo Fisher Scientific). Results are the average of the two reproducible experiments.

### 3.7. Statistical Analysis

One way ANOVA followed by post hoc comparison using least significant difference (LSD) and independent *t*-test were performed for data comparison using SPSS 18.0 statistical software. Significant difference was *p* < 0.05. 

## 4. Conclusions

Human single chain antibodies (HuscFvs) to *S. aureus* TSST-1 that inhibited the TSST-1-mediated T cell activation and pro-inflammatory cytokine gene expressions and productions were generated. The HuscFvs formed interface contact with the TSST-1 residues important for immunopathogenesis of toxic shock syndrome. The HuscFvs have high potential for testing further as a direct acting anti-TSST-1 agent for future clinical use. 

## Figures and Tables

**Table 1:** LC-MS/MS Mascot results of peptides generated from recombinant TSST-1 of this study with 30% sequence coverage.

Proteins	Orthologous Protein	Accession No.	Number of Matched Peptides	Score	Matched Peptide Sequence (Score)
TSST-1	Toxic shock syndrome toxin-1 of *S. aureus*	Gi 136457	9	239	DSPLKYGPK (44)
					LPTPIELPLKVK (38)
					HQLTQIHGLYR (36)
					ITMNDGSTYQSDLSK (91)
					ITMNDGSTYQSDLSK (35)
					ITMNDGSTYQSDLSK (28)
					ITMNDGSTYQSDLSK (28)
					NTDGSISLIIFPSPYYSPAFTKGEK (32)
					NTDGSISLIIFPSPYYSPAFTKGEK (23)

**Table 2:** TSST-1 residues and motives predicted to be bound by residues and domains ofHuscFv35. After residues labeled in red were mutated to alanines, the HuscFv35 lost ability to suppress TSST-1 mitogenicity and pyrogenicity.

TSST-1		HuscFv35		Intermolecular Bond
Residue	Motif	Residue(s)	Domain	
S1	α1-helix	T165	VL-CDR1	Van de Waals
D4	Before α1-helix	T165	VL-CDR1	Van de Waals
D4	Before α1-helix	N166	VL-CDR1	H-bond
I6	α1-helix	N166	VL-CDR1	H-bond
K7	α1-helix	Y168	VL-CDR1	Water bridge
K7	α1-helix	Y227	VL-CDR3	H-bond
K7	α1-helix	D228	VL-CDR3	H-bond
D8	α1-helix	Y103	VL-CDR3	OH-π
D8	Before α1-helix	N166	VL-CDR1	Van de Waals
W12	α1-helix	L102	VH-CDR3	CH-π
S15	α1-helix	Q101	VL-CDR3	H-bond
G16	Before β1-strand	Q101	VH-CDR3	Hydrophobic
K67	β4-strand	Q101	VH-CDR3	Water bridge
R68	β4-strand	D31	VH-CDR1	H-bond
R68	β4-strand	H100	VH-CDR3	CH-π
R68	β4-strand	Q101	VH-CDR3	Van de Waals
K71	β4-strand	H100	VH-CDR3	CH-π
K71	β4-strand	D108	VH-CDR3	H-bond
K71	β4-strand	Y185	VL-FR2	H-bond
S72	β4-strand	H100	VH-CDR3	H-bond
Q73	β4-strand	T192	VL-FR3	H bond
Y80	β5-strand	Y27	VH-FR1	H bond
Y80	β5-strand	D31	VH-CDR1	OH-π

**Table 3:** TSST-1 residues and motives predicted to be bound by residues and domains of HuscFv53. After residues labeled in red were mutated to alanines, the HuscFv53 lost ability to suppress TSST-1 mitogenicity and pyrogenicity.

TSST-1		HuscFv53		Intermolecular Bond
Residue	Motif	Residue(s)	Domain	
D18	Before β1-strand	R31	VH-CDR1	H-bond
D39	Before β3-strand	R31	VH-CDR1	H-bond
R68	β4-strand	T52	VH-CDR2	Van de Waals
R68	β4-strand	D57	VH-CDR2	H-bond
K71	β4-strand	W33	VH-CDR1	NH-π
K71	β4-strand	T52	VH-CDR2	H-bond
K71	β4-strand	D57	VH-CDR2	H-bond
Q73	β4-strand	W33	VH-CDR1	Van de Waals
Q73	β4-strand	W230	VL-CDR3	Van de Waals
H74	β4-strand	W33	VH-CDR3	π-stacking
H74	β4-strand	R100	VH-CDR3	NH-π
H74	β4-strand	F101	VL-CDR1	π-stacking
S76	Before β5-strand	D166	VL-CDR1	H bond
S76	Before β5-strand	Y168	VL-CDR1	H bond
S76	Before β5-strand	K186	VL-CDR2	H bond
E77	Before β5-strand	K186	VL-CDR2	Water bridge
Y80	β5-strand	W33	VH-CDR1	H-bond
Y80	β5-strand	R100	VH-CDR3	NH-π

**Table 4:** TSST-1 residues and motives predicted to be bound by residues and domains ofHuscFv56. After residues labeled in red were mutated to alanines, the HuscFv56 lost ability to suppress TSST-1 mitogenicity and pyrogenicity.

TSST-1		HuscFv56		Intermolecular Bond
Residue	Motif	Residue(s)	Domain	
K7	α1-helix	S162	VL-CDR1	H-bond
K7	α1-helix	I163	VL-CDR1	Van der Waals
L10	α1-helix	I163	VL-CDR1	Hydrophobic
D11	α1-helix	I163	VL-CDR1	Van de Waals
D11	α1-helix	R164	VL-CDR1	H-bond
D11	α1-helix	Y165	VL-CDR1	OH-π
S15	α1-helix	Y165	VL-CDR1	H-bond
S17	Before β1-strand	Y102	VL-CDR3	H-bond
D18	Before β1-strand	R103	VH-CDR3	Water bridge
T19	β1-strand	Y102	VH-CDR3	CH-π
T19	β1-strand	R103	VH-CDR3	Van de Waals
F20	β1-strand	R103	VH-CDR3	CH-π
D39	Before β3-strand	Y182	VL-FR2	Water bridge
D39	Before β3-strand	P189	VL-CDR2	Van de Waals
N65	β4-strand	Y102	VH-CDR3	OH-π
R68	β4-strand	Y182	VL-FR2	H bond
R68	β4-strand	N186	VL-CDR2	Van de Waals
K71	β4-strand	S185	VL-CDR2	Van de Waals
K71	β4-strand	N186	VL-CDR2	Van de Waals
S72	β4-strand	N186	VL-CDR2	H bond
H74	β4-strand	F193	VL-FR3	π-stacking
Y80	Β5-strand	Y182	VL-FR2	π-stacking
Y80	Β5-strand	N186	VL-CDR2	Van de Waals
Y80Y80	Β5-strand	V187	VL-CDR2	H bond
	Β5-strand	F193	VL-FR3	π-stacking
K114	Before β8-strand	S57	VH-CDR2	Van de Waals
K114K114	Before β8-strand	T58	VH-CDR2	H bond
	Before β8-strand	E59	VH-CDR2	Salt bridge
Y115	Before β8-strand	W50	VH-CDR2	π-stacking
Y115Y115	Before β8-strand	F52	VH-CDR2	π-stacking
	Before β8-strand	Y101	VH-CDR3	H-bond
P117	Before β8-strand	F52	VH-CDR2	CH-π
P117	Before β8-strand	Y101	VH-CDR3	CH-π
P117	Before β8-strand	Y102	VH-CDR3	CH-π
K118	β8-strand	S31	VH-CDR1	H bond
K118	β8-strand	E55	VH-CDR2	H bond
K118	β8-strand	Y102	VH-CDR3	H bond
F119	β8-strand	Y102	VH-CDR3	π-stacking
E132	α2-helix	Y101	VH-CDR3	OH-π
E132	α2-helix	Y102	VH-CDR3	CH-π
E132	α2-helix	R104	VH-CDR3	Salt bridge
H135	α2-helix	R104	VH-CDR3	NH-π
H135	α2-helix	W224	VL-CDR3	π-stacking
Q139	α2-helix	W224	VL-CDR3	CH-π
Q139	α2-helix	Y227	VL-CDR3	CH-π
Q139	α2-helix	Y229	VL-CDR3	H bond
I140	α2-helix	Y227	VL-CDR3	CH-π
R145	Before β9-strand	S225	VL-CDR3	H-bond

## Supplementary Materials

The following are available online at www.mdpi.com/2072-6651/9/2/50/s1, Figure S1: Codon-optimized nucleotide and deduced amino acid sequences of the TSST-1 of this study. Figure S2: Effect of lipopolysaccharide (LPS) on PBMC stimulation compared to TSST-1. Figure S3: Binding of the purified, refolded HuscFv35 (T35), HuscFv53 (T53) and HuscFv56 (T56) to rTSSt-1 and SEA immobilized on the ELISA well surface with and without BSA. Figure S4: Computerized bindingof TSST-1 (cyan) and HuscFvs (green). Figure S5: Residues and motives of TSST-1. Table S1: Estimated accuracy of the modeled HuscFv35, HuscFv53, and HuscFv56. Table S2: Oligonucleotide primers used in quantitative real-time RT-PCR for monitoring cytokine gene expressions.
