# Supplementary material for: Human scFvs That Counteract Bioactivities of Staphylococcus aureus TSST-1
Source: Toxins (Basel). 2017 Feb 17;9(2):50. doi: 10.3390/toxins9020050 (PMC5331430; doi:10.3390/toxins9020050)
Supplement: Supplementary file 1 [file toxins-09-00050-s001.pdf]

Supplementary Materials: Human scFvs that counteract bioactivities of *Staphylococcus aureus* TSST-1

Thunchanok Rukkawattanakul[1,2], Nitat Sookrung[2,3], Watee Seesuay[2], Nattawat Onlamoon[3], Pornphan Diraphat[4], Wanpen Chaicumpa[2] and Nitaya Indrawattana[5,*]

A

```
       GGATCC
  7:   TCGACGAATGACAACATCAAAGACCTGCTGGACTGGTACTCCTCGGGCTCGGATACGTTC
 61:   ACGAATAGCGAAGTGCTGGACAACTCACTGGGTAGCATGCGTATCAAAAATACCGATGGT
127:   AGCATTAGCCTGATCATCTTTCCGTCACCGTATTACAGCCCGGCATTCACCAAAGGCGAA
187:   AAAGTGGATCTGAATACCAAACGCACGAAAAAATCACAGCATACCTCAGAAGGTACCTAC
247:   ATCCACTTTCAGATCAGCGGCGTGACCAACACCGAAAAACTGCCGACCCCGATTGAACTG
307:   CCGCTGAAAGTGAAAGTTCATGGCAAAGATTCGCCGCTGAAATATGGTCCGAAATTTGAT
367:   AAAAAACAGCTGGCAATTTCGACCCTGGATTTCGAAATTCGCCACCAGCTGACCCAGATC
427:   CATGGTCTGTACCGTTCAAGCGACAAAACCGGCGGTTATTGGAAAATCACCATGAATGAT
487:   GGTTCGACGTACCAGAGCGATCTGTCGAAAAAATTCGAATACAACACGGAAAAACCGCCG
547:   ATTAATATCGATGAAATCAAAACCATCGAAGCGGAAATCAAT
       AAGCTT
```

B

```
  1:   STNDNIKDLLDWYSSGSDTFTNSEVLDNSLGSMRIKNTDGSISLIIFPSPYYSPAFTKGE
 61:   KVDLNTKRTKKSQHTSEGTYIHFQISGVTNTEKLPTPIELPLKVKVHGKDSPLKYGPKFD
121:   KKQLAISTLDFEIRHQLTQIHGLYRSSDKTGGYWKITMNDGSTYQSDLSKKFEYNTEKPP
181:   INIDEIKTIEAEIN
```

Figure S1. (A) Codon-optimized nucleotide sequence of the TSST-1 of this study. (B) The deduced amino acid sequence of the rTSST-1 which has 100% amino acid identity to the TSST-1 of the database (accession J02615).

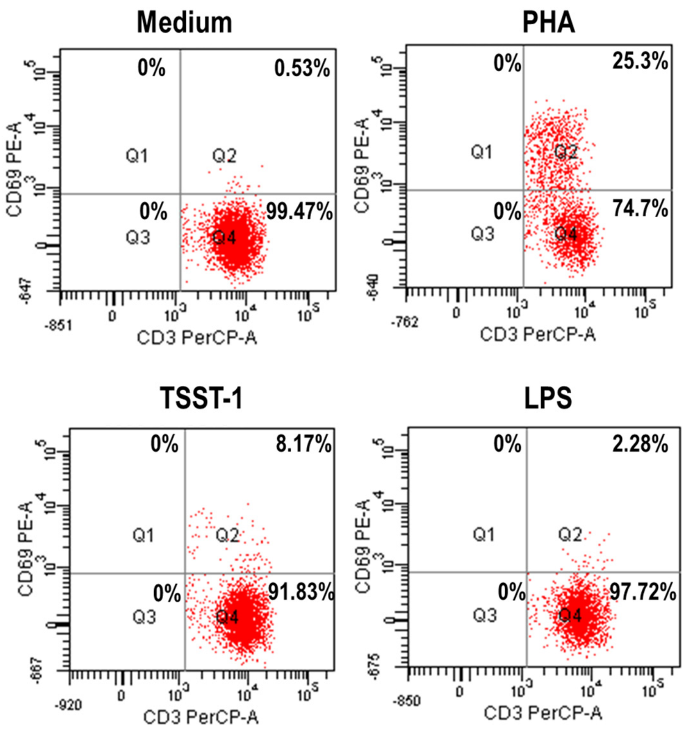

Figure S2. Effect of lipopolysaccharide (LPS) on PBMC stimulation compared to TSST-1. TSST-1 at 1000 ng/mL induced proliferation of 8.17% of the cells while the LPS induced proliferation of 2.28% of the cells (28% of the proliferated cells induced by TSST-1).

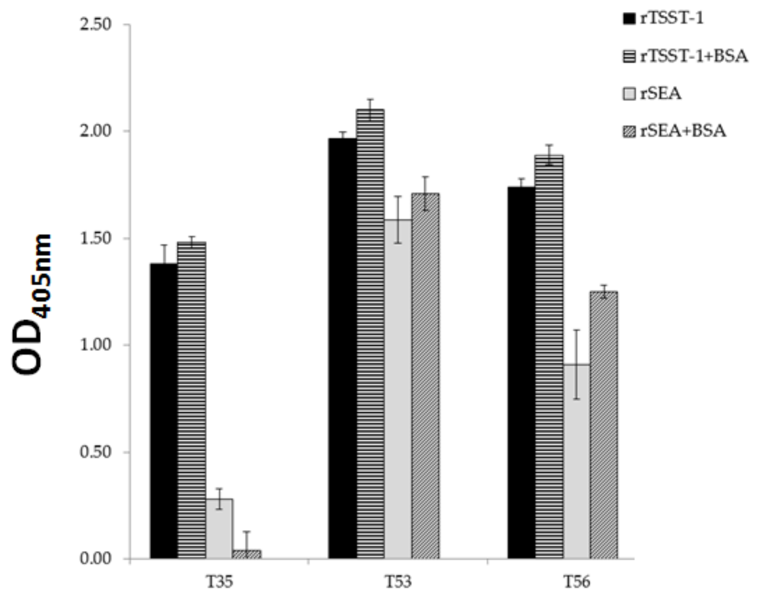

Figure S3. Binding of the purified, refolded HuscFv35 (T35), HuscFv53 (T53) and HuscFv56 (T56) to rTSSt-1 and SEA immobilized on the ELISA well surface with and without BSA.

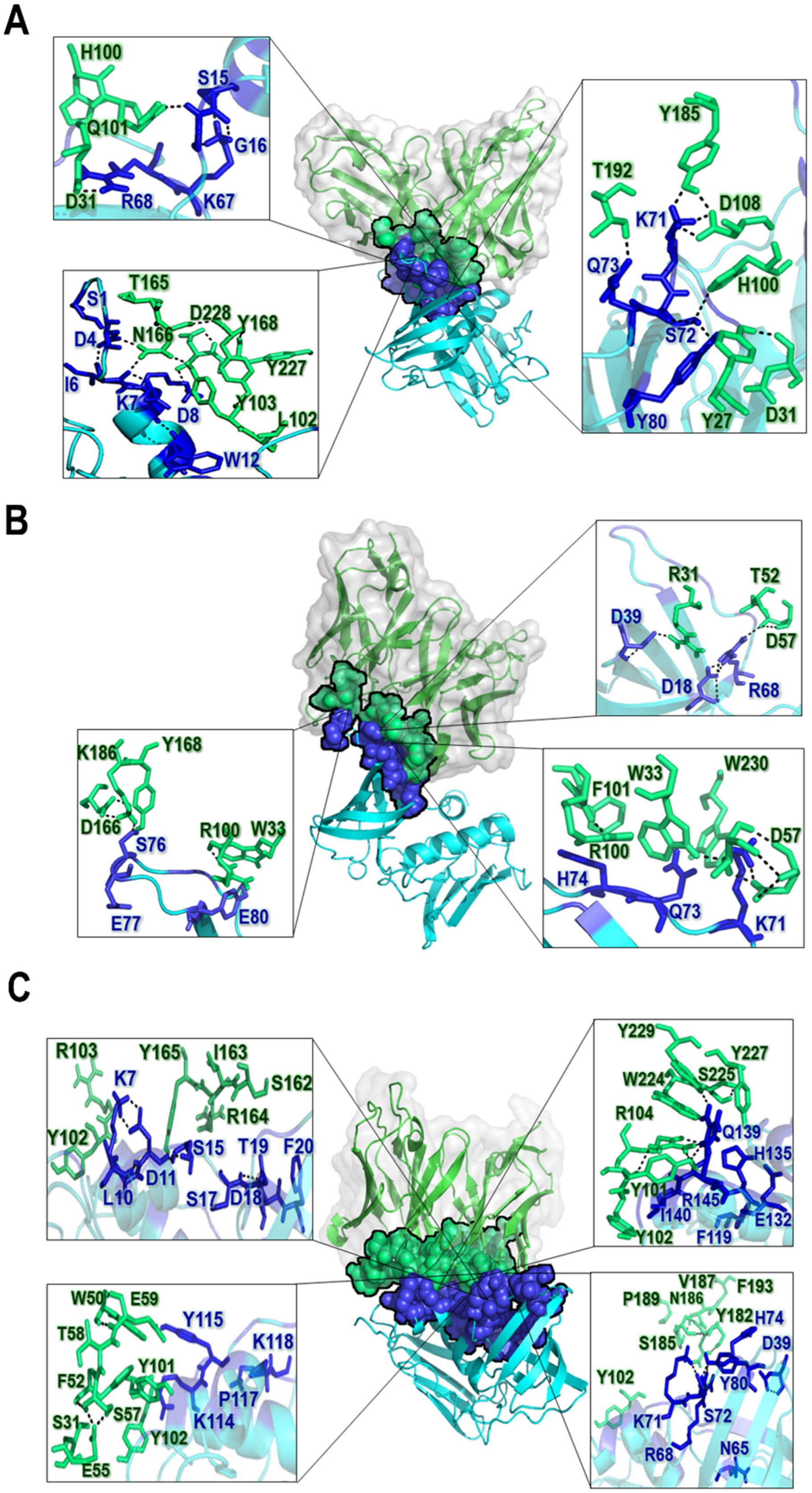

Figure S4. Computerized binding of TSST-1 (cyan) and HuscFvs (green): (A)-(C) complexes of TSST-1 with HuscFv35, HuscFv53, and HuscFV56, respectively. Enlarged square areas show interactive residues between TSST-1 (blue) and HuscFvs (green).

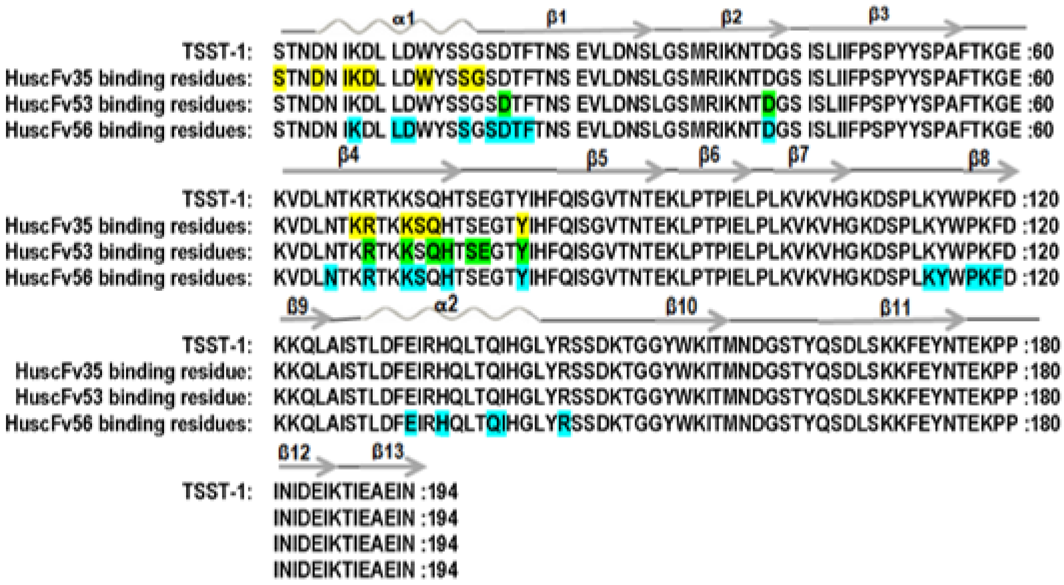

Figure S5. Residues and motives of TSST-1. The residues that formed interface contact with HuscFv35, HuscFv53, and HuscFv56 are colored in yellow, green, and cyan, respectively.

Table S1. Estimated accuracy of the modeled HuscFv35, HuscFv53, and HuscFv56

Protein name	C-score	TM-score	RMSA(A˚)	No. of decoys	Cluster density
HuscFv35	0.91	0.84±0.08	3.9±2.6	9659	0.6032
HuscFv53	0.96	0.84±0.08	3.8±2.6	9718	0.6526
HuscFv56	1.15	0.87±0.07	3.4±2.4	9395	0.7676

Table S2. Oligonucleotide primers used in quantitative real-time RT-PCR for monitoring cytokine gene expressions.

Gene encoding	Primer	Size of product (bp)
**IL-1β**	F: 5'- CCAGCTACGAATCTCCGACC-3'	142
	R: 5'- GGGAAAGAAGGTGCTCAGGT-3'	
**IL-6**	F: 5'- CCAGTACCCCCAGGAGAAGA-3'	112
	R: 5'- CTGAGATGCCGTCGAGGATG-3'	
**TNFα**	F: 5′- AGCTGCCCCTCAGCTTGA-3′	85
	R: 5′- CCCAGGCAGTCAGATCATCTT-3′	
**β-actin**	F: 5′- CATCGAGCACGGCATCGTCA-3′	208
	R: 5′- TAGCACAGCCTGGATAGC-3′	
